# Correction: An innovative chalcogenide transfer agent for improved aqueous quantum dot synthesis

**DOI:** 10.1039/d4sc90237h

**Published:** 2024-12-06

**Authors:** Guillaume Petit, Cedric Malherbe, Pauline Bianchi, Jean-Christophe M. Monbaliu

**Affiliations:** a Center for Integrated Technology and Organic Synthesis (CiTOS), MolSys Research Unit, University of Liège B-4000 Liège (Sart Tilman) Belgium jc.monbaliu@uliege.be; b Mass Spectrometry Laboratory, MolSys Research Unit, University of Liège B-4000 Liège (Sart Tilman) Belgium; c WEL Research Institute Avenue Pasteur 6 B-1300 Wavre Belgium

## Abstract

Correction for ‘An innovative chalcogenide transfer agent for improved aqueous quantum dot synthesis’ by Guillaume Petit *et al.*, *Chem. Sci.*, 2024, **15**, 13148–13159, https://doi.org/10.1039/D4SC01135J.

The authors regret that in the original article, some references to prior literature were incorrectly listed in Fig. 1a and the optional zinc additive for the CdSe/ZnS core–shell quantum dots (QDs) was mislabelled in Fig. 1b.

The corrected version of Fig. 1 is provided here.

**Fig. 1:**
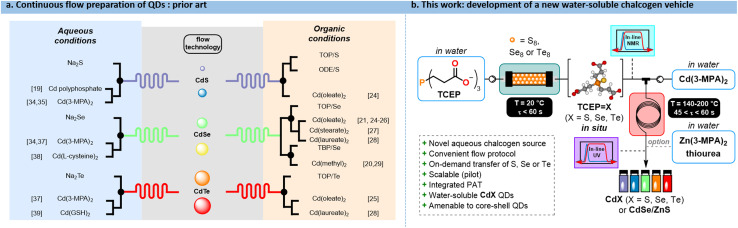
(a) Protocols from the prior Art for accessing QDs with flow processes. For each chalcogen (S, Se, Te), the precursors are summarized according to the reaction medium (organic/aqueous). (b) This work reports a fully concatenated flow process in water for accessing **CdX** (X = S, Se, Te) QDs, as well as **CdSe**/**ZnS** core–shell QDs.

The Royal Society of Chemistry apologises for these errors and any consequent inconvenience to authors and readers.

